# Deregulation of Genes Related to Iron and Mitochondrial Metabolism in Refractory Anemia with Ring Sideroblasts

**DOI:** 10.1371/journal.pone.0126555

**Published:** 2015-05-08

**Authors:** Mónica del Rey, Rocío Benito, Celia Fontanillo, Francisco J. Campos-Laborie, Kamila Janusz, Talía Velasco-Hernández, María Abáigar, María Hernández, Rebeca Cuello, Daniel Borrego, Dionisio Martín-Zanca, Javier De Las Rivas, Ken I. Mills, Jesús M. Hernández-Rivas

**Affiliations:** 1 IBMCC, Centro de Investigación del Cáncer (CIC), Universidad de Salamanca-CSIC, Salamanca, Spain; 2 IBSAL, Instituto de Investigación Biomédica de Salamanca, Salamanca, Spain; 3 Instituto de Biología Funcional y Genómica, CSIC-Universidad de Salamanca, Salamanca, Spain; 4 Servicio de Hematología, Hospital Clínico de Valladolid, Valladolid, Spain; 5 Centre for Cancer Research and Cell Biology, Queen’s University Belfast, Belfast, United Kingdom; 6 Servicio de Hematología, Hospital Universitario de Salamanca, Salamanca, Spain; University of Florida, UNITED STATES

## Abstract

The presence of *SF3B1* gene mutations is a hallmark of refractory anemia with ring sideroblasts (RARS). However, the mechanisms responsible for iron accumulation that characterize the Myelodysplastic Syndrome with ring sideroblasts (MDS-RS) are not completely understood. In order to gain insight in the molecular basis of MDS-RS, an integrative study of the expression and mutational status of genes related to iron and mitochondrial metabolism was carried out. A total of 231 low-risk MDS patients and 81 controls were studied. Gene expression analysis revealed that iron metabolism and mitochondrial function had the highest number of genes deregulated in RARS patients compared to controls and the refractory cytopenias with unilineage dysplasia (RCUD). Thus mitochondrial transporters *SLC25* (*SLC25A37* and *SLC25A38*) and *ALAD* genes were over-expressed in RARS. Moreover, significant differences were observed between patients with *SF3B1* mutations and patients without the mutations. The deregulation of genes involved in iron and mitochondrial metabolism provides new insights in our knowledge of MDS-RS. New variants that could be involved in the pathogenesis of these diseases have been identified.

## Introduction

Myelodysplastic syndromes (MDS) are clonal hematological disorders characterized by blood cytopenias, ineffective hematopoiesis and hypercellular bone marrow [1]. According to the WHO classification (2008), six subtypes of MDS are distinguished: refractory cytopenia with unilineage dysplasia (RCUD), refractory anemia with ring sideroblasts (RARS), refractory cytopenia with multilineage dysplasia (RCMD), refractory anemia with excess blasts (RAEB-1 and RAEB-2), MDS-unclassified (MDS-U) and MDS associated with isolated del(5q) [2]. Patients with RARS present with isolated anemia, hypochromic erythrocytes, hyperplastic ineffective erythropoiesis and mitochondrial ferritin accumulation in erythroid precursor cells. RARS and RCMD with ring sideroblasts (RCMD-RS) are defined by the presence of more than 15% ringed sideroblasts. The accumulation of ferritin is present in the ring sideroblasts and this is likely to be involved in the increased apoptosis of erythroblasts and, therefore, in ineffective erythropoiesis [3].

Iron is essential for heme synthesis and Fe-S cluster biogenesis in the erythroid cell. Iron is acquired by the erythroid precursors and it is imported into mitochondria by SLC25A37 (Mitoferrin-1) [4]. This protein is a member of the solute carrier family, and is localized in the inner mitochondrial membrane, where it is an essential iron importer. Heme synthesis is initiated in the mitochondrion and the iron that is not incorporated during this process is stored in Fe-S clusters and transported out of the mitochondrion by the ABCB7 membrane protein [5,6,7].

High expression of some heme biosynthesis-related genes, such as *ALAS2* and *FECH*, have been seen in RARS patients [8], whilst low levels of *ABCB7* gene expression in patients with RARS compared with other MDS subtypes have also been found [9]. However, no mutations in these genes have been detected in acquired RARS [9,10]. By contrast, several genetic lesions have been identified in inherited sideroblastic anemias, including mutations in the *SLC25A38*, *ALAS2* and *ABCB7* genes [11,12,13,14,15].

The presence of recurrent somatic mutations of the splicing factor 3B subunit 1 (*SF3B1*) gene in a high proportion of patients with RARS (64–83%) or RCMD-RS (57–76%) have been recently demonstrated [16,17,18,19,20,21]. *SF3B1* is located on chromosome 2q33.1 and encodes the SF3B1 protein, which plays a role in pre-mRNA splicing and associated transcription [21]. In addition, recent studies have shown a possible role of *SF3B1* in the formation of ring sideroblasts in MDS [22,23]. However, some authors suggest that RARS is a disease resulting from a specific alteration in one or more genes involved in mitochondrial function, iron distribution, or both [3]. Thereby, the abnormal mitochondrial iron metabolism that characterizes RARS is not completely understood and the pathogenesis of ring sideroblasts in MDS remains to be clarified.

In order to gain insight in our knowledge of the abnormal iron accumulation, defective mitochondrial function and ineffective heme biosynthesis in low-risk MDS, an integrative study of both expression and mutational status of genes related to iron and mitochondria was carried out. Our study has shown that *SLC25A37* and *SLC25A38* were over-expressed in RARS patients, and has identified one sequence change in the *ALAD* gene that could contribute to a better understanding of the pathogenesis of sideroblastic MDS.

## Materials and Methods

### Patients, samples and cell separation

A total cohort of 231 low-risk MDS patients and 81 controls were included in the studies performed in this work. 69 low-risk MDS patients (30 RARS patients and 39 RCUD patients) and 31 controls without hematological malignancies were included in the gene expression profiling study. Moreover, 6 MDS with ring sideroblasts were analyzed by massive DNA-sequencing techniques, while 175 low-risk MDS patients were analyzed by conventional Sanger sequencing (100 MDS with ring sideroblasts and 75 were other low-risk MDS) (S1 Table). All patients were classified according to the World Health Organization (WHO) 2008 criteria [2] (with the exception of RCMD-RS, which we maintain from the World Health Organization, 2002 [24] as a separate category). In addition, 50 healthy controls were also included in the Sanger sequencing study. Mononuclear cells were isolated from bone marrow (BM) of MDS patients and controls by density gradient (Ficoll). The unfractionated mononuclear cells were used for the expression and sequencing studies. In addition, CD3+ cells from peripheral blood from patients of interest were purified using magnetically activated cell sorting (MACS) CD3 MicroBeads (Miltenyi Biotec, Bergisch Gladbach, Germany). The study was approved by the Local Ethical Committee “Comité Ético de Investigación Clínica, Hospital Universitario de Salamanca” and written informed consent was obtained from each patient and their relatives.

### RNA and DNA isolation

Total RNA was extracted from cells by homogenization in TRIZOL (Invitrogen, Carlsbad, CA, USA) following the manufacturer’s protocol, treated with RQ1 RNAse-Free DNase (Promega, Madison, USA) to eliminate genomic DNA contamination, and finally purified with RNeasy Minikit (Qiagen, Hilden, Germany). RNA quantity and quality was determined with an Agilent 2100 Bioanalyzer (Santa Clara, CA, USA). Genomic DNA from subject samples was isolated using DNeasy blood and tissue kit following the manufacturer’s protocol (Qiagen).

### Gene expression microarray studies

Gene expression profiling (GEP) studies were carried out as part of the Microarray Innovations in LEukemia (MILE) study [25]. GeneChip Human Genome U133 plus 2.0 arrays (Affymetrix, High Wycombe, UK) are gene expression arrays containing 54 613 oligonucleotide probesets that map onto 18 950 human gene loci (obtained using the mapping of microarrays probes to ENSEMBL gene IDs provided by GATExplorer) [26]. RNA was labeled and hybridized according to protocols from Affymetrix. Briefly, 100 ng of total RNA was amplified and labeled using the GeneChip two-cycle cDNA synthesis kit and GeneChip IVT labeling kit (Affymetrix Inc.) and then hybridized onto a Human Genome U133 Plus 2.0 microarray, after quality checking on GeneChip Test3 arrays. Washing and scanning were done with a Fluidics Station 400 and GeneChip Scanner (Affymetrix Inc.) as previously described [27]

#### Bioinformatic methods for global expression profiling and differential analysis

Robust Microarray Analysis (RMA) algorithm was applied to the raw data of the expression arrays to achieve background correction and intra- and inter-normalization, and to calculate the expression signal [28]. The Significant Analysis of Microarrays (SAM) algorithm was used to identify genes with statistically significant changes differences in expression between different classes [29]. For this purpose, samples were permuted over 100 cycles by using the two-class (unpaired) and multiclass response format, assuming unequal variances for the genes. Significant genes were selected on the basis of a false discovery rate (FDR) of <0.05. To select each gene the p-values of each statistical test were transformed to q-values using the indicated FDR threshold. The algorithms described were applied using the R programming environment and the Bioconductor package.

#### Validation of the gene expression signature with independent cohort of patients

In order to validate the gene expression signatures obtained with the MDS patient cohorts studied, an independent analysis was performed using gene expression data from bone marrow CD34+ cells of MDS patients and healthy controls obtained from GSE19429 (GEO database: http://www.ncbi.nlm.nih.gov/geo/).

#### Differential analysis in low-risk MDS with and without *SF3B1/SRSF2* mutations

In order to find genes that mark possible differences between low-risk MDS samples with mutated *SF3B1/SRSF2* genes (n = 22) and low-risk MDS samples without mutations (n = 13), a recursive algorithm written in R was designed and applied. This alogarithm produces multiple subsets of samples from each class and run differential expression analysis for each one of these subsets using LIMMA [30]. In this way, random selected groups of 7 *versus* 7 samples were analyzed running a total of 10000 differential expression tests. The recursive algorithm identified and counted the genes that gave a significant differential expression in each one of the contrasts (using as cutoff: q-value < 0.01, that corresponds to the p-value adjusted by FDR). In this way, a gene signature of 200-top genes was selected. Once these genes were selected we used an outcome/response algorithm called Global Test [31] to check further if the genes selected were able to predict the two classes investigated.

The same strategy was used to compare MDS-RS with mutated *SF3B1* (n = 13) and non-mutated *SF3B1* (n = 6). However in this case, we random selected groups of 4 *versus* 4 samples running a total of 7425 differential expression analyses and a p-value adjusted by FDR < 0.10 was used. A gene signature of 75-top genes was selected, being genes that appeared as significant in at least ten different contrasts.

#### Functional enrichment analysis

To analyze the functional enrichment of the selected gene lists the bioinformatic resource DAVID (http://david.abcc.ncifcrf.gov/) [32] and the web-delivered bioinformatics tool set IPA (Ingenuity Pathway Analysis 9.0; http://www.ingenuity.com) were used. Both tools enable the functional modules and the most relevant biological processes present in the gene lists to be identified by statistical enrichment analysis based on contingency tests.

### Targeted Sequence Capture and DNA Sequencing assay

Array-based sequence capture (Roche NimbleGen) followed by next-generation sequencing (Roche GS FLX Titanium sequencing platform) was used to analyze 93 genes related to hematological malignancies. Details of the design of the array, 454 sequencing, coverage statistics and data analysis are provided in the Supplementary Methods (S1 Methods).

### Real-Time PCR

The expression levels of selected genes were analyzed by Real-Time PCR. First-strand cDNA was generated from 1 μg of total RNA using poly-dT as primer and the M-MLV reverse transcriptase (Promega). Real-time PCR was performed in triplicate and was analyzed as previously described [33]. The primers were designed for specific sequences (S2 Table) and checked by the BLAST algorithm [34].

### Sanger sequencing

To elucidate and validate the presence of possible genes variations, Sanger re-sequencing was carried out. Oligonucleotide primers were designed against all exons of *SLC25A37* and a genomic fragment of exons 6 and 7 in the *ALAD* gene. A pair of primers to amplify a 1 696-bp genomic fragment and a second reverse internal primer for the sequencing in *ALAD* was used. All primers were designed using Primer3 (http:/frodo.wi.mit.edu/primer3/) (S3 Table). In addition, the previously published primers against the exons more frequently mutated of *SLC25A38* in congenital sideroblastic anemia [14] and against the exon 14 and 15 of *SF3B1* were used [17]. Genomic DNA was amplified with the Fast Start High Fidelity PCR System (Roche, Basel, Switzerland) following the manufacturer’s instructions and including some variations of the annealing temperature and magnesium concentration (S3 Table). DNA sequences were evaluated using Scanner v1.0 (Applied Biosystems, Carlsbad, CA, USA) and Accelrys DS Gene v1.5 software. Data were analysed using annotations of genome version GRCh37 (hg19).

#### Bioinformatics analysis of missense variants

The effects of amino acid changes on protein function were predicted with SIFT using the protein sequences of human ALAD as the input. Homologous protein sequences of the human *ALAD* gene were retrieved from the NCBI genome database with BLASTP.

## Results

### Gene expression analysis reveals an iron-related profile in RARS patients

The gene expression profile (GEP) from the BM of 30 RARS patients was compared with that of 31 healthy individuals. A total of 1 145 genes showed significant differences (q-value<0.05) in mRNA expression levels between the two groups: 700 and 445 genes were over and under-expressed, respectively, in the RARS samples (S1 Fig and S4 Table). The most over-expressed gene in RARS was *GDF15* (p-value < 0.0001; R fold = 20.27). In addition, genes associated with iron and mitochondrial metabolism represented the largest function group of genes involved: 38% (266 molecules) of the over-expressed genes were identified in this study. Of these genes, 106 were related to mitochondria function, 13 were related to iron binding, including *CYBRD1*, *STEAP3* and *ACO1*, while 11 were involved in the heme biosynthetic process, six of which (*ALAD*, *HMBS*, *UROS*, *UROD*, *CPOX*, *PPOX*) had a direct role in heme formation. In addition, *ABCB6*, whose function is to move coproporphyrinogen III from the cytoplasm into the mitochondria during heme biosynthesis, was over-expressed in RARS patients. Nine genes were related to cellular iron ion homeostasis, of which *TF*, *TFR2*, *TFRC*, *FXN*, *SLC25A37* and *SCL25A38* were up-regulated in RARS patients (Table 1).

**Table 1:** Most representative deregulated cellular functions in RARS patients respect to the control group.

*Fuction*	*Number of genes*	*p Value*	*Over-expressed genes*
MITOCHONDRION	106	5,70658E-46	
Membrane and Mitochondrion	32	0,00000000	*VDAC3*, *NDUFV3*, *SLC25A15*, *SLC25A36*, *SLC25A38*, *ATP7B*, *BCS1L*, *C18orf55*, *HAX1*, *TIMM50*, *NDUFB6*, *APOO*, *HTRA2*, *CYB5A*, *MTX2*, *NDUFS2*, *TMEM14C*, *ABCB6*, *SLC25A37*, *C3orf1*, *CCDC90A*, *ABCB10*, *STOML2*, *STXBP1*, *HK1*, *NDUFS4*, *MRPS17*, *PPOX*, *TIMM44*, *MAOA*, *SLC25A20*, *CLIC4*
Mitochondrial inner membrane	23	0,00000000	*VDAC3*, *CHCHD3*, *NDUFV3*, *SLC25A15*, *SLC25A36*, *SLC25A38*, *DCI*, *NME4*, *BCS1L*, *NDUFB6*, *COX15*, *APOOL*, *NDUFS2*, *SLC25A37*, *SHMT2*, *HSPD1*, *FDXR*, *ABCB10*, *GCDH*, *STOML2*, *GCAT*, *NDUFS4*, *SLC25A20*
Cytoplasm and Mitochondrion	21	0,00000002	*LONP1*, *GART*, *PPP2CA*, *ATP7B*, *CAPRIN2*, *APOO*, *CYB5A*, *TP53*, *DARS2*, *TXN2*, *HSPD1*, *HEBP1*, *STOML2*, *SOD1*, *STXBP1*, *ISOC2*, *THG1L*, *AIFM1*, *OAT*, *CLIC4*, *HMBS*
Mitochondrial matrix	16	0,00000003	*DNAJA3*, *DCI*, *ETFA*, *COQ3*, *ACAT1*, *MIPEP*, *PCCB*, *DARS2*, *HSPD1*, *FDXR*, *FXN*, *GCDH*, *SOD1*, *HSPE1*, *TIMM44*, *OAT*
Nucleus and Mitochondrion	14	0,00000128	*GART*, *PPP2CA*, *C14orf156*, *HAX1*, *TIMM50*, *REXO2*, *HTRA2*, *TP53*, *APEX2*, *BRD8*, *TSFM*, *SOD1*, *ISOC2*, *AIFM1*
Nucleolus and Mitochondrion	4	0,00077681	*GART*, *TP53*, *TXN2*, *BRD8*
PROTEIN BINDING	25	0,00000000	*VDAC3*, *CHCHD3*, *LONP1*, *DLAT*, *GART*, *MRPS9*, *PPP2CA*, *ATP7B*, *PMAIP1*, *TIMM50*, *ACAT1*, *TUFM*, *SHMT2*, *FDXR*, *FXN*, *GCDH*, *STXBP1*, *ISOC2*, *TIMM44*, *ATP5B*, *ARG2*, *MAOA*, *AIFM1*, *OAT*, *CLIC4*
NUCLEOTIDE BINDING	17	0,00000000	*VDAC3*, *LONP1*, *GART*, *ATP7B*, *NME4*, *C14orf156*, *BCS1L*, *TRAP1*, *DARS2*, *TUFM*, *ABCB6*, *HSPD1*, *ABCB10*, *MRPL39*, *HK1*, *TIMM44*, *ATP5B*
ATP BINDING	15	0,00000006	*LONP1*, *GART*, *ATP7B*, *NME4*, *BCS1L*, *TRAP1*, *TP53*, *DARS2*, *ABCB6*, *HSPD1*, *ABCB10*, *HSPE1*, *HK1*, *TIMM44*, *ATP5B*
TRANSPORT	15	0,00000027	*NDUFV3*, *SLC25A15*, *SLC25A36*, *SLC25A38*, *ETFA*, *NDUFB6*, *CYB5A*, *NDUFS2*, *TXN2*, *ABCB6*, *FDXR*, *ABCB10*, *NDUFS4*, *MRPS17*, *SLC25A20*
IRON ION BINDING	13	0,00003287	*PPAT*, *RRM2*, *PPP2CA*, *LIAS*, *DOHH*, *RFESD*, *CYBRD1*, *MIPEP*, *ACO1*, *SLC11A2*, *NDUFS2*, *SLC25A37*, *STEAP3*
ELECTRON CARRIER ACTIVITY	13	0,00028086	*ETFA*, *RFESD*, *NDUFS2*, *TXN2*, *TSTA3*, *FDXR*, *STEAP3*, *GCDH*, *TXNL3*, *PPOX*, *MAOA*, *AIFM1*, *SUOX*
HEME BIOSYNTHETIC PROCESS	11	0,00000001	*ALAD*, *UROS*, *FXN*, *PPOX*, *CPOX*, *UROD*, *HMBS*, *COX15*, *EPRS*, *BLVRB*, *ABCB6*
ELECTRON TRANSPORT CHAIN	11	0,00000514	*NDUFV3*, *FADS2*, *ETFA*, *CYBRD1*, *NDUFB6*, *CYB5A*, *TXNL1*, *NDUFS2*, *TXN2*, *FDXR*, *NDUFS4*
CELLULAR IRON ION HOMEOSTASIS	9	0,00000053	*MYC*, *TF*, *ABCB6*, *FXN*, *TFR2*, *SOD1*, *TFRC*, *SLC25A37*, *SLC25A38*
HYDROLASE ACTIVITY	8	0,00009485	*MTHFD2*, *PPP2CA*, *ATP7B*, *REXO2*, *HINT2*, *THEM2*, *ATP5B*, *ARG2*
METABOLIC PROCESS	8	0,00017248	*DLAT*, *C5orf33*, *DCI*, *LIAS*, *ATP7B*, *COQ3*, *ACAT1*, *ISOC2*
OXIDOREDUCTASE ACTIVITY	8	0,00062130	*RFESD*, *TSTA3*, *FDXR*, *STEAP3*, *PPOX*, *MAOA*, *AIFM1*, *SUOX*
ACYLTRANSFERASE ACTIVITY	6	0,04443590	*DLAT*, *ESCO2*, *KS*, *NAT13*, *GCAT*, *TGM2*
ION TRANSPORT	5	0,00039908	*ATP7B*, *TF*, *SLC25A37*, *ATP5B*, *CLIC4*

To determine whether the genes involved in mitochondrial metabolism were exclusive to the RARS expression profile, this group of patients was compared with other low-risk MDS cases (RCUD group). The comparative analysis of the gene expression profile of both groups identified a set of 192 differentially expressed genes: 128 genes were up-regulated in RARS patients while 64 were down-regulated (S2 Fig and S5 Table). Interestingly, 33% (42 genes) of the over-expressed genes were related to iron and mitochondrial metabolism as inferred from the functional enrichment analyses. 33 genes were related to mitochondria, five were associated with iron binding and eight were involved in heme formation. Thus, *ALAD*, *HMBS*, *UROS*, *UROD*, *CPOX* and *PPOX* were over-expressed in RARS patients with respect to the other low-risk MDS (Table 2).

**Table 2:** Most representative deregulated cellular functions in RARS patients respect to the RCUD group.

*Fuction*	*Number of genes*	*p Value*	*Over-expressed genes*	
MITOCHONDRION	33	0,000000		
Integral to membrane	15	0,047316	*GBGT1*, *ST6GALNAC4*, *SLC25A38*, *AADACL1*, *CYBRD1*, *TSPAN17*, *RHBDD1*, *APOO*, *TMEM14C*, *KCNH2*, *MS4A7*, *PPAPDC1A*, *STEAP3*, *ABCB10*, *AC079061*.*8*
Cytoplasm and mitochondrion	7	0,000042	*ATP7B*, *CAPRIN2*, *APOO*, *HSPD1*, *HEBP1*, *ISOC2*, *HMBS*
Membrane	6	0,002383	*SLC25A38*, *ATP7B*, *APOO*, *TMEM14C*, *ABCB10*, *PPOX*
Mitochondrial inner membrane	4	0,009138	*SLC25A38*, *NME4*, *HSPD1*, *ABCB10*
HEME BIOSYNTHETIC PROCESS	8	0,000000	*ALAD*, *UROS*, *PPOX*, *CPOX*, *UROD*, *HMBS*, *EPRS*, *BLVRB*	
NUCLEOTIDE BINDING	6	0,000019	*ATP7B*, *NME4*, *C14orf156*, *TRAP1*, *HSPD1*, *ABCB10*	
ATP BINDING	5	0,000078	*ATP7B*, *NME4*, *TRAP1*, *HSPD1*, *ABCB10*	
PROTEIN BINDING	5	0,002258	*MRPS9*, *ATP7B*, *ACAT1*, *ISOC2*, *ARG2*	
IRON ION BINDING	5	0,000516	*PPAT*, *PIR*, *RFESD*, *CYBRD1*, *STEAP3*	

#### Validation of the RARS expression signature with independent datasets

In order to validate the expression signatures found in our unfractionated cohort of MDS samples, a comparison between the present studies and an independent cohort of MDS patients where CD34+ cells were isolated (published by Pellagatti *et al*. 2010) [35] was carried out. Odds ratio was used to measure how strongly is the overlapping of the altered genes in both analysis. Analysing the Pellagatti *et al*. dataset (GSE19429), a total of 5 840 genes showed significant differences (FDR ≤ 0.05) in mRNA expression levels between CD34+ cell from RARS patients (using only MDS-RS samples with normal karyotype) and their control group: 3 697 and 2 143 genes were over and under-expressed, respectively, in the RARS samples. This signature had a significant overlap of 457 genes with our signature for the same RARS vs controls comparison (only considering the up-regulated genes); which corresponds to an odds ratio for this comparison of 7.14 (Lower CI 95% = 8.38).

Further analysis of the Pellagatti data (34) for the CD34+ differential expression between RARS and RCUD groups identified a set of 404 differentially expressed genes (FDR ≤ 0.05): 284 genes were up-regulated in RARS patients while 120 were down-regulated. A very significant overlap was also observed between this up-regulated gene set and the set of 128 genes that we discovered up-regulated in our RARS patients versus the RCUD group (Odds ratio = 31.83; Lower CI 95% = 21.51).

### A targeted genome capture and next-generation sequencing strategy identifies gene variants in MDS with ring sideroblasts

In order to identify any gene variants in MDS-RS, a capture and sequence approach was done on 93 genes from a group of 6 MDS with ring sideroblasts using a custom NimbleGen array was carried out: 39 of the gene targets were related to iron and mitochondrial metabolism (S6 Table). The enrichment assay followed by NGS detected a total of 8 230 variants in all patients analyzed (median 1 367 variants per sample, range 1 078–1 672). All putative variants were first compared with published single nucleotide polymorphism (SNP) data from dbSNP130. The 2 217 known SNPs (56%) were discarded along with the new variants found at non-coding regions (85%) and, finally, those that did not give rise to an amino-acid change in their protein sequence (58%)

As a result, a missense variation was detected in the *ALAD* gene in one case with ring sideroblasts (Chr9: 116,152,735); therefore the incidence of this variation, and known SNPs, were examined, by Sanger sequencing, in a larger cohort of 100 MDS patients with ring sideroblasts. In addition, conventional mutational analysis as alternative approach was also performed for the *SLC25A37* and *SLC25A38* genes.

### RARS patients present a frequent haplotype in exon 6 and a new variant in exon 7 of the ALAD gene

Two known polymorphisms have been reported in exon 6 of the *ALAD* gene: rs8177807 (T10728C) and rs2228083 (C10679T). The SNPs were located at positions Chr9: 116,152,891 and Chr9: 116,152,940, respectively, 49 base pairs from each other. The study revealed two possible haplotypes for these variants: “common haplotype” (T10728 and C10679) and “variant haplotype” (C10728 and T10679). Interestingly, the “variant haplotype” was present in 12 of 100 MDS with ring sideroblasts (12%), while it was only found in 5 of 100 controls and RCUD patients (5%) (p = 0.07).

**Fig 1:**
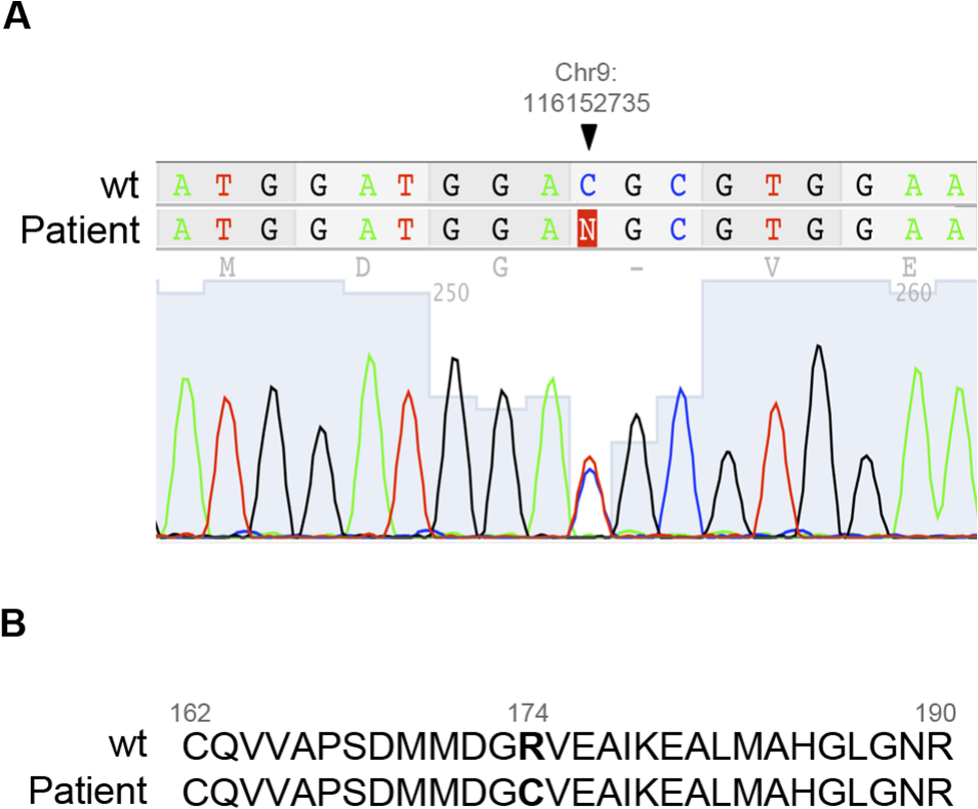
Variation in the *ALAD* gene in a RARS case. **(A)** New missense variation in the *ALAD* gene in the BM of one RARS patient found by massive sequencing. The variation is heterozygous and is located at Chr9: 116,152,735 position in exon 7. **(B)** Protein sequences from wild-type and RARS patient. Amino-acid change in the protein sequence of ALAD in a RARS patient. Arginine (R174) is replaced by cysteine y the mutant protein. (RARS: refractory anemia with ring sideroblasts)

**Fig 2:**
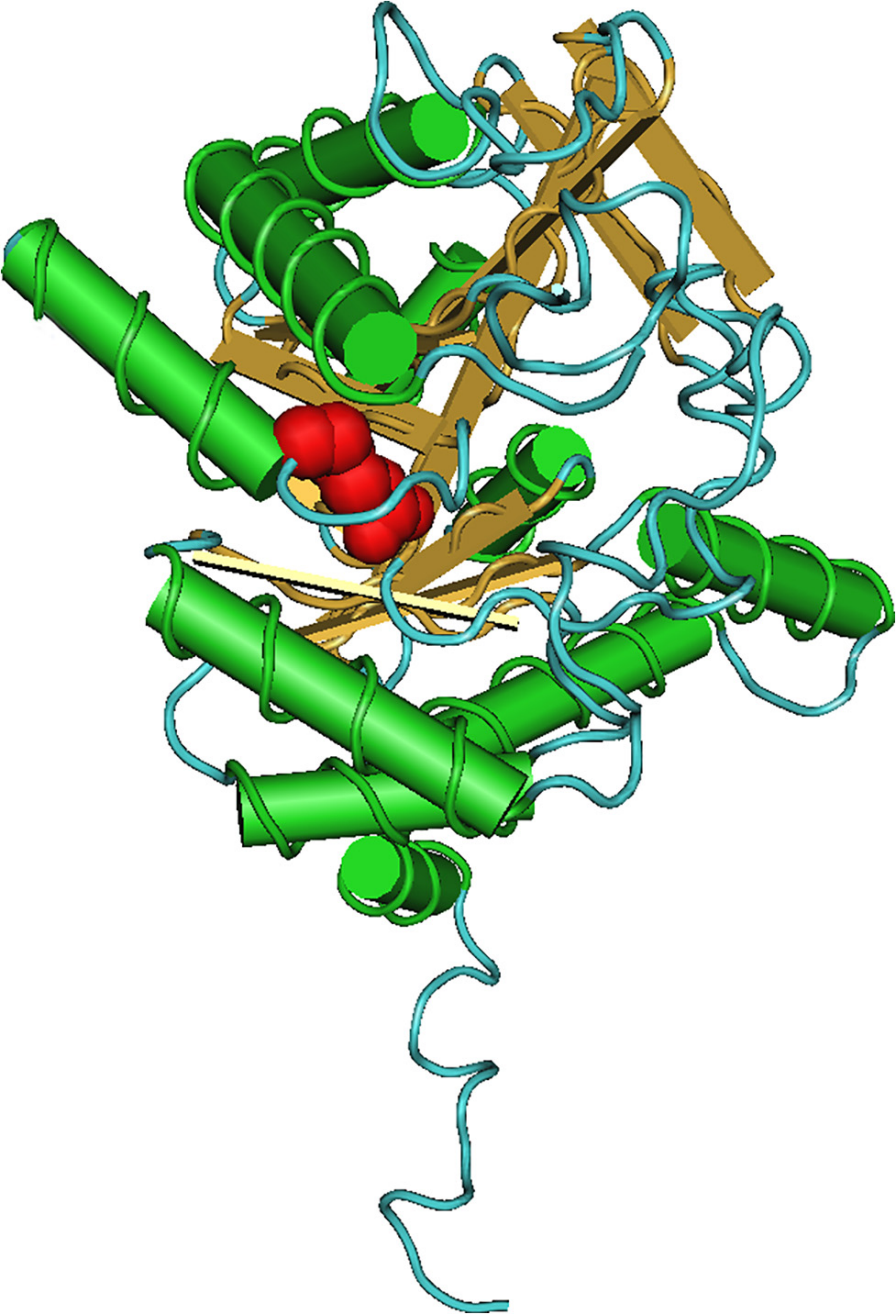
Three-dimensional structure of the ALAD protein. Monomeric structure of the ALAD protein. The red color indicates the position of the arginine 174. The amino acid is completely buried into the monomeric structure of the ALAD protein.

In addition, exon 7 of the *ALAD* gene was analyzed in a larger cohort of MDS with ring sideroblasts (n = 100) and the change previously identified by the capture and sequence approach was confirmed by conventional sequencing (Fig 1A). The positively charged arginine residue (R174) was replaced by an uncharged cysteine residue (Fig 1B). The three-dimensional structure showed that R174 residue is completely buried into the monomeric structure (Fig 2) and the protein was predicted to be potentially damaging.

Furthermore, the variant was found in the CD3+ cells of PB from the same patient suggesting the presence of a germ line mutation. The variant was not found in any of the control samples or in those analysed from RCUD patients.

### *SLC25A37* (Mitoferrin-1) is un-mutated in acquired RARS

*SLC25A37*, that encodes a mitochondrial iron transporter that specifically mediates iron uptake in developing erythroid cells, was over-expressed in RARS patients (R Fold = 1.70). Therefore, a complete sequence analysis of the *SLC25A37* gene was performed in the group of 50 patients MDS with ring sideroblasts: 62% of all the cases analyzed had at least one known polymorphism in their sequence. Specifically, the polymorphisms were located in exons 2 and 4 of *SLC25A37* with 6% of the patients having the rs1047384 polymorphism (exon 4). The majority of polymorphisms (56%) were in exon 2; furthermore, 38%, 14% and 4% of cases exhibited one, two or three polymorphisms in this exon, respectively (S3 Fig). All polymorphisms were homozygous. No new variations were observed in the *SLC25A37* gene in the patients with ring sideroblasts.

### *SLC25A38* showed a new mutation in one patient with RCUD

**Fig 3:**
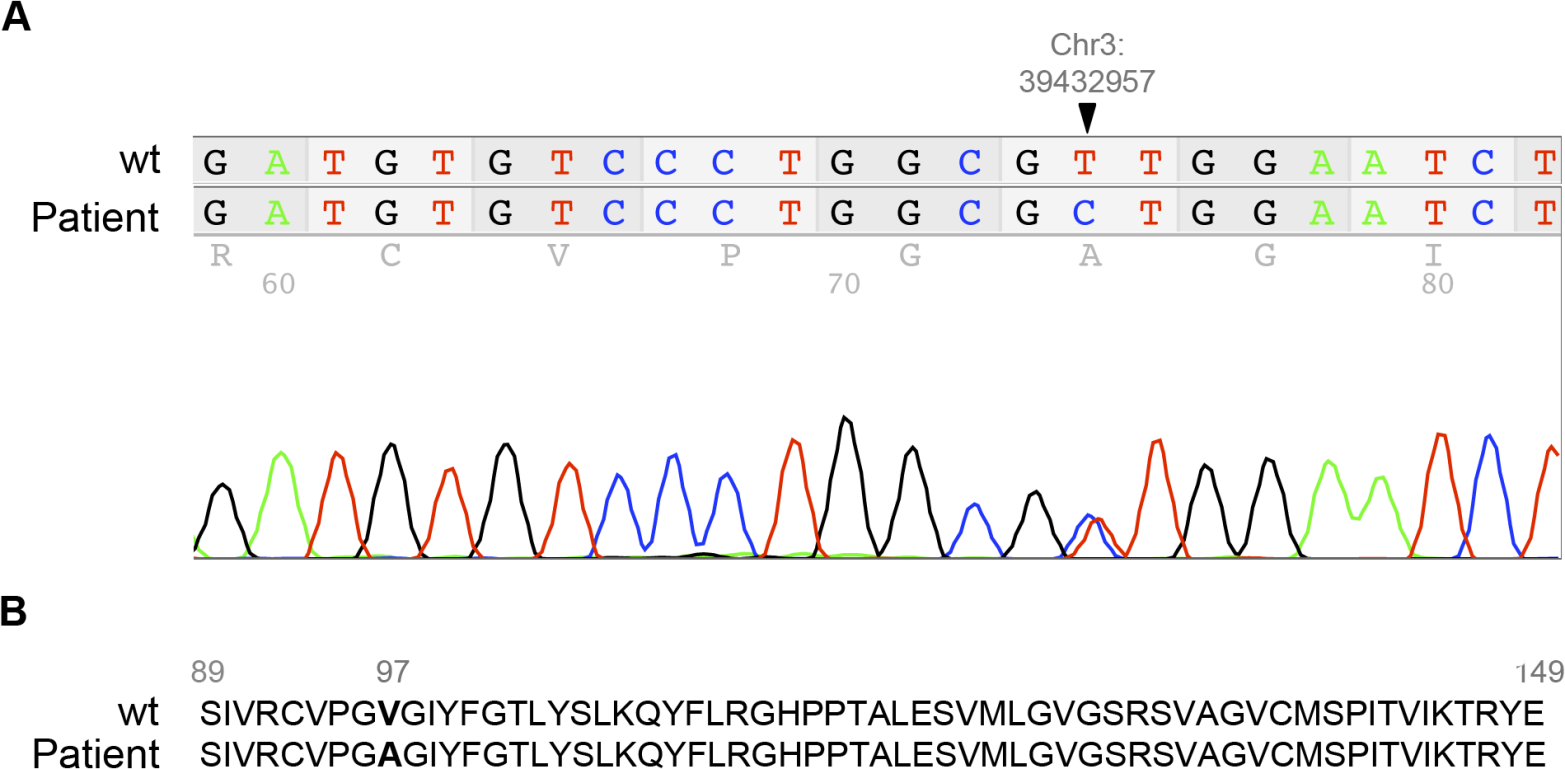
*SLC25A38* mutation in a RCUD patient. (A) New missense mutation in exon 4 of a RCUD patient detected in BM by Sanger sequencing. The mutation is heterozygous and is located at the Chr3: 39,432,957 position. (B) Amino-acid change in the protein sequence of SLC25A38 in the RCUD patient. Valine 97 is replaced by alanine in the mutant protein. (RCUD: refractory cytopenia with unilineage dysplasia).

*SCL25A38*, recently described as a mutated gene in congenital sideroblastic anemia, was found to be over-expressed in RARS patients in our study (R Fold = 1.78). We further analyzed this gene by sequencing in the group of low-risk MDS patients (n = 175). A new missense mutation was observed in the BM from one of the RCUD patients. The mutation, in exon 4, was located at position Chr3: 39,432,957 and resulted in the amino-acid change of valine for alanine (V97A) (Fig 3A–3B).

In addition, three known polymorphisms (rs1995236, rs870843 and rs9877539) were observed in the *SCL25A38* sequence in the group of low-risk MDS. They were located close to the coding sequence for 4, 6 and 7 exons. Interestingly, both rs1995236 and rs9877539 polymorphisms were homozygous for the less common variants in the population (100% and 97.6% of all the cases with these variations, respectively) (S7 Table).

### Recurrent *SF3B1* and *SRSF2* Mutations in low-risk MDS patients

*SF3B1*, recently described as a mutated gene in a high proportion of MDS patients with ring sideroblasts, was analyzed in the same cohort of patients with MDS-RS (n = 100). *SF3B1* was mutated in 82 of 100 cases with ring sideroblasts (82%). Exon 15 was more frequently mutated (47%) than exon 14 (32%). The mutations in exon 15 were located in the same codon (700) while those in exon 14 were located in four codons: 622 (7%), 625 (1%), 662 (12%) and 666 (12%). All patients had a single mutation in the gene, except for three cases (3%), which had two changes. One of them showed the mutations in the same exon (codons 662 and 666) while the mutations in the other two patients were located in different exons (codons 666 and 700). However, the clinical characteristics of these patients did not differ from those with a single mutation.

No relationships between the presence of polymorphisms in *SCL25A37* or *SLC25A38* and *SF3B1* mutations were found. The case with *ALAD* variation showed a mutation in the *SF3B1* gene (exon 14, codon 662).

In addition, a mutational study of *SF3B1* and *SRSF2* in all RCUD patients with available expression profile information was carried out. 30% of the cases showed mutations in *SF3B1*, 10% carried variations in *SRSF2* and 60% showed non-mutations in any of the genes analyzed. The mutations detected in RCUD were located in hot-spots previously described by other groups. Specifically, they were observed in codons 622, 625, 662, 666 and 700 in the *SF3B1* gene and codon 95 in *SRSF2*.

### Gene expression profile showed significant differences between mutated patients and non-mutated cases

A supervised analysis of GEP between mutated low-risk MDS cases (RARS and RCUD) and non-mutated low-risk MDS patients was carried out. Herein, *GDF15* was the most up-regulated gene in low-risk MDS patients with mutation in spliceosome-related genes. *ALAD*, *SLC25A37*, *SLC11A2*, *PCK2*, *MRPS9*, *AIFM2* and *HK1*, all of them involved in iron and mitochondrial metabolism, were also over-expressed and were included in the Top200 more differentially expressed genes (S8 Table). In addition, a functional study with this gene Top200 gene set showed that cell cycle (Benjamini-Hochberg value < 0.0003) and mitosis (Benjamini-Hochberg value < 0.005) were the most frequently deregulated molecular functions, involving 24 and 21 differentially expressed genes, respectively.

A supervised analysis between the gene expression levels of *SF3B1* mutations patients and non-mutated MDS-RS cases was carried out. An over-expressed gene-signature of 71 genes was identified between both sub-groups (S9 Table). Interestingly, *GDF15* was overexpressed in patients showing *SF3B1* mutations. In addition, other genes such as *PPP2R5B*, *PPP1R16A* and *DDIT4L*, related to *SF3B1* and *GDF15*, were up-regulated in the mutated group. A functional analysis with this gene set showed two deregulated pathways: porphyrin biosynthesis and heme biosynthesis (p< 0.001). *ALAS2*, *PPOX* and *UROD*, all of them involved in heme formation, were also over-expressed in the mutated patients.

## Discussion

The integrative analysis of the massive gene analysis has provided new insights in the knowledge of the pathogenesis of MDS [27]. In the present study, an over-expression of iron and mitochondrial metabolism related genes was observed in patients with RARS. Therefore a custom sequence capture array was designed in order to identify genes that could play a role in the pathogenesis of MDS with ring sideroblasts.

The use of unfractionated compared to fractionated cells in this type of expression or sequencing study has become a matter of debate in recent years. The approach taken in the present study is supported by previous studies that have been able to identify biological and prognostic characteristics in MDS and AML by studying the gene expression profile and the use of unfractionated mononuclear cells [27,36,37]. Furthermore, the results of the present study are supported by previous reports in CD34+ cells [8] due to a significant overlap between both analysis. The use of CD34+ cells is of great scientific value while the analysis of unfractionated samples could allow the identification of the interaction between the different cell types. In this respect, the present study has suggested that the erythroid lineage displays a robust gene expression signature that allows the identification from the global set of mononuclear cells.

Interestingly, the most significant functional category from the gene expression signature was iron and mitochondrial metabolism. This category had the highest proportion of genes over-expressed in RARS patients when compared to the controls or the RCUD group, representing 38% and 33% of all over-expressed genes, respectively. Of note, six key enzymes in the heme biosynthesis pathway showed increased expression, some members of this pathway are also over-expressed in CD34+ cells of RARS patients [8]. In addition to the enzymes that catalyze heme formation, our study highlighted the over-expression of the *ABCB6* gene in RARS patients. This molecule is ideally located in the outer membrane, where it can move coproporphyrinogen III from the cytoplasm into the mitochondrion using ATP hydrolysis as the source of energy [7,38]. The deregulation of *ABCB6* expression may contribute to the impaired heme biosynthesis found in MDS with ring sideroblasts.

The *ALAD* gene encodes a cytosolic enzyme that catalyses the condensation of two molecules of d-aminolevulinic acid (ALA) to form porphobilinogen (PBG) in the second step of the heme biosynthetic pathway [39]. Our analysis of the ALAD gene identified two polymorphisms in exon 6 located 49 bases from each other and, interestingly, the presence of one of them was always determined by the presence of the other one. The occurrence of both polymorphisms (“variant haplotype”) was more frequent in MDS with ring sideroblasts (12%) than in members of the other groups analyzed (5%). Therefore, our study showed a trend to an association of the variant haplotype with the ring sideroblasts. The presence of haplotypes has been linked to the deregulation of the genes in different hematological malignances, such as chronic lymphocytic leukemia and acute lymphoblastic leukemia [40,41]. Thus, these findings could be related to the deregulation of the *ALAD* gene and consequently to the abnormal iron and mitochondrial metabolism in MDS with ring sideroblasts.

The capture and sequencing study identified a non-described sequence change in the *ALAD* gene in one RARS patient showing a *SF3B1* mutation; the *ALAD* gene was also up-regulated in the gene expression studies. The variants of this gene were also found in the CD3+ population in the PB. In addition, the variant led to amino-acid change in the ALAD protein. Therefore this variant could have a possible role in the predisposition to disease (as a first event) as well as contributing to the pathogenesis of RARS where *SF3B1* mutations are the trigger cause.

In addition, we have identified a new missense mutation in exon 4 of the *SCL25A38* gene in one RCUD patient. Thus, the mutation led to the 97V>A amino-acid change in the protein sequence. *SLC25A38* gene has been previously linked to congenital sideroblastic anemia [14]. These findings would indicate that mutations in the *SLC25A38* gene might be associated to the low-risk MDS.

*SLC25A37* is a member of the mitochondrial solute carrier family. Some authors have shown that SLC25A37 contributes to mitochondrial iron acquisition in mammalian cells, since decreases in *SLC25A37* severely reduce mitochondrial iron-consuming processes, such as Heme and Fe-S cluster synthesis. In fact, mitochondrial iron transport is reduced by more than 90% in cells silenced for SLC25A37, suggesting that SLC25A37 is a major contributor to mitochondrial iron acquisition [42,4]. Our results showed up-regulation of *SLC25A37* in RARS patients with respect to the control group. These findings led us to hypothesize that this over-expression could be responsible of iron accumulation. For these reasons, we sought out to determine whether MDS with ring sideroblasts cases were characterized by *SLC25A37* somatic mutations. No mutations were found for this gene in cases with ring sideroblasts. Therefore, other mechanisms give rise to the over-expression of the *SLC25A37* gene in RARS patients. In addition, it should be noted that no relationships between the presence of polymorphisms in *SCL25A37* or *SLC25A38* and *SF3B1* mutations were found.

The GEP from the BM of *SF3B1* mutated patients was compared to that from the BM of non-mutated individuals. 71 genes showed over-expression in mRNA levels in mutated cases. Interestingly, two pathways observed in the functional analysis were related to mitochondrial metabolism. *GDF15*, a cytokine from the TGFβ family, was one of the most highly differentially expressed gene. *GDF15* is expressed at high levels in patients with ineffective erythropoiesis. In contrast to the low levels of *GDF15* expressed during normal erythropoiesis, ineffective erythropoiesis causes high-level expression of *GDF15* [43]. In addition, it has been suggested that over-expression of *GDF15* in patients with RARS might be involved in the systemic iron overload by suppressing hepcidin secretion [43,44], being sensitive to iron depletion and this response is specifically antagonized by the reprovision of iron [45]. Therefore, the *GDF15* over-expression could be related to the presence of mutations in *SF3B1* gene, and therefore, to a higher percentage of ring sideroblasts previously described [17]. These findings also suggest that the up-regulation of *ALAS2*, *PPOX* and *UROD*, all of them involved in heme formation, could be related to the presence of *SF3B1* mutations. Furthermore, the comparison between the GEP of mutated low-risk MDS patients and non-mutated cases showed the cell cycle and mitosis as the most frequently deregulated pathways. This would support the hypothesis that a mutation in spliceosome-related genes could be the trigger cause, and support the presence of a second event for the deregulation of iron and mitochondrial metabolism.

In summary, our integrated expression and sequencing approaches has identified both the deregulation of genes involved in iron and mitochondrial metabolism and a new variant in the *ALAD* gene. Both potential mechanisms provide new insights into the pathogenesis of MDS with ring sideroblasts and, specifically, in patients with *SF3B1* mutations.

## Supporting Information

S1 Fig Graphical representation of the differentially expressed genes between RARS patients and control group. 700 genes were over-expressed and 445 genes were under-expressed in the RARS cases. Each point represents the log2 of R.fold value from each gene.
(PDF)

S2 Fig Graphical representation of the differentially expressed genes between RARS and RCUD patients. 128 genes were up-regulated and 64 were down-regulated in RARS cases. Each point represents the log2 of R.fold value from each gene.
(PDF)

S3 Fig Graphical representation of the polymorphisms present in exon 2 of the *SLC25A37* gene. 56% of the analyzed cases had some polymorphism in exon 2. The patients showed one, two or three polymorphisms in this exon in 38%, 14% and 4% of the cases respectively. The most common polymorphism was rs2942194 as isolated variation. The analysis showed two possible combinations for the patients with two polymorphisms: rs2942194 and rs10992 or rs10992 and rs3736032. The first combination was more frequent than the second combination. The combination between rs2942194 and rs3736032 was not found in any patient.
(TIF)

S1 Methods (DOC)

S1 Table Clinical and biological characteristics of low-risk MDS patients. (XLS)

S2 Table Primers sequences designed for Real-Time PCR. (XLSX)

S3 Table Primers sequences designed for Sanger Sequencing. (XLSX)

S4 Table Over-expressed and under-expressed genes in RARS patients respect to the control group. (XLSX)

S5 Table Over-expressed and under-expressed genes in RARS patients respect to the RCUD group. (XLSX)

S6 Table Iron and mitochondrial genes targeted by sequence capture. (XLSX)

S7 Table Presence of polymorphisms in SLC25A38 in low-risk MDS patients. (XLSX)

S8 Table Top200 gene-signature from mutated low-risk MDS patients respect to non-mutated low-risk MDS cases. (XLSX)

S9 Table Over-expressed gene-signature from SF3B1 mutated patients respect to non-mutated cases. (XLSX)
