# Supplementary figures and images for: Deregulation of Genes Related to Iron and Mitochondrial Metabolism in Refractory Anemia with Ring Sideroblasts
Source: PLoS One. 2015 May 8;10(5):e0126555. doi: 10.1371/journal.pone.0126555 (PMC4425562; doi:10.1371/journal.pone.0126555)

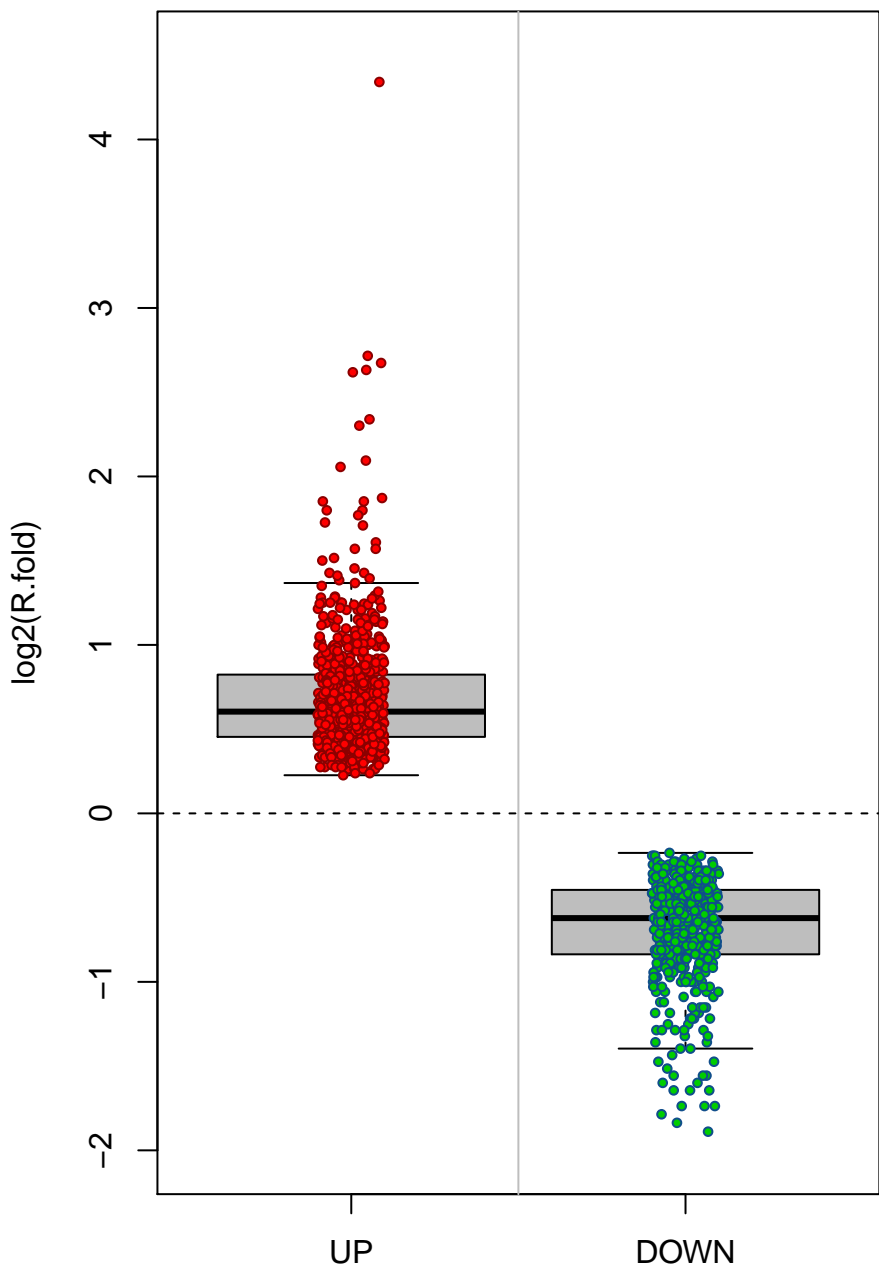

Supplement: S1 Fig — 700 genes were over-expressed and 445 genes were under-expressed in the RARS cases. Each point represents the log2 of R.fold value from each gene. (PDF) [file pone.0126555.s001.pdf]

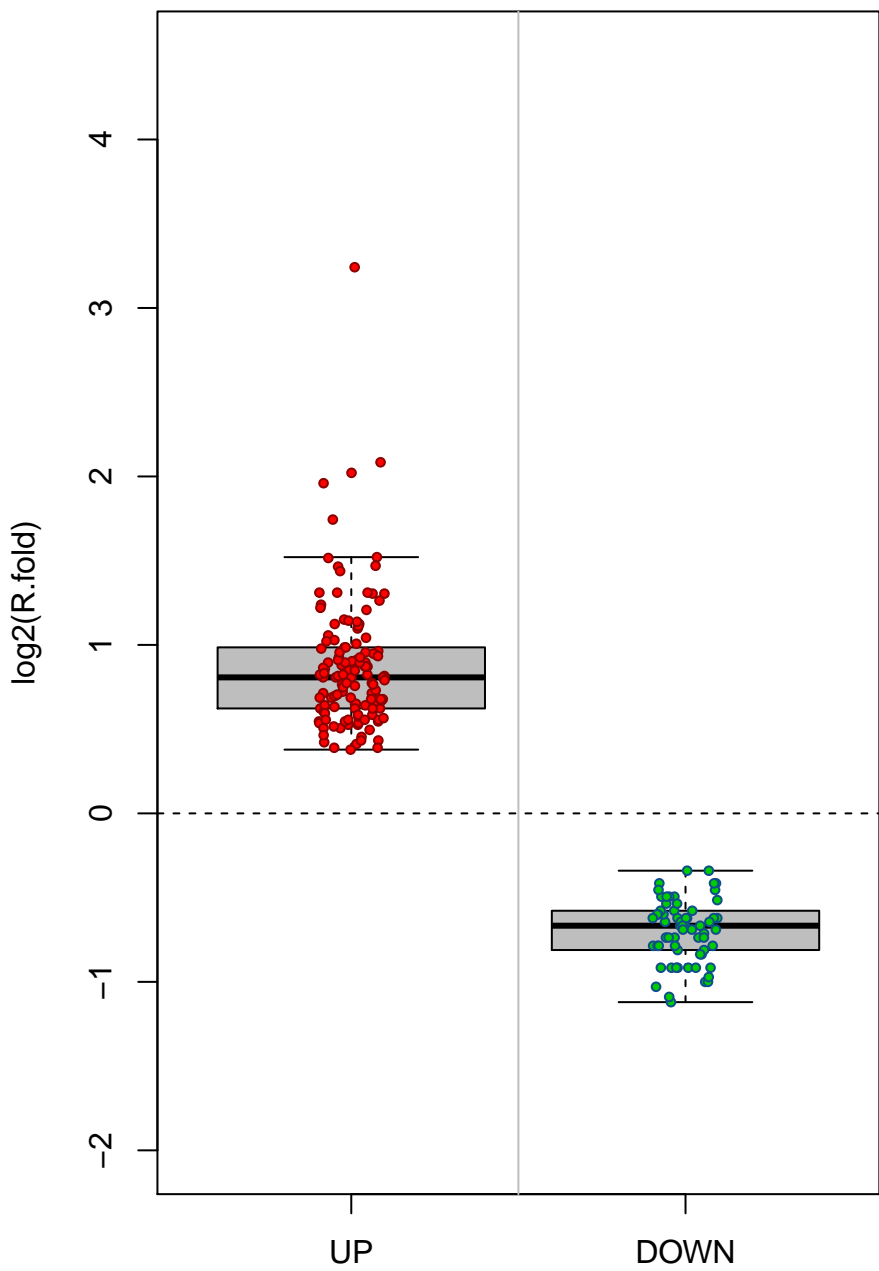

Supplement: S2 Fig — 128 genes were up-regulated and 64 were down-regulated in RARS cases. Each point represents the log2 of R.fold value from each gene. (PDF) [file pone.0126555.s002.pdf]

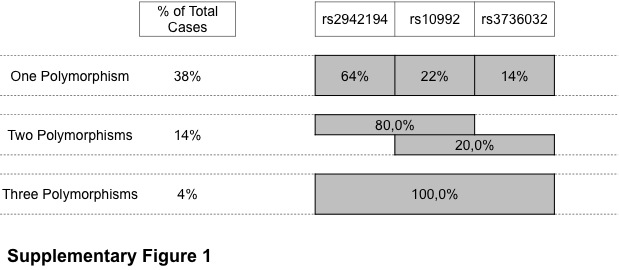

Supplement: S3 Fig — 56% of the analyzed cases had some polymorphism in exon 2. The patients showed one, two or three polymorphisms in this exon in 38%, 14% and 4% of the cases respectively. The most common polymorphism was rs2942194 as isolated variation. The analysis showed two possible combinations for the patients with two polymorphisms: rs2942194 and rs10992 or rs10992 and rs3736032. The first combination was more frequent than the second combination. The combination between rs2942194 and rs3736032 was not found in any patient. (TIF) [file pone.0126555.s003.tif]
