# Supplementary material for: Deregulation of Genes Related to Iron and Mitochondrial Metabolism in Refractory Anemia with Ring Sideroblasts
Source: PLoS One. 2015 May 8;10(5):e0126555. doi: 10.1371/journal.pone.0126555 (PMC4425562; doi:10.1371/journal.pone.0126555)
Supplement: S1 Methods — (DOC) [file pone.0126555.s004.doc]

**Supplementary Methods**

**NimbleGen Target-Region Capture**

A custom Sequence Capture 385K Human Array was designed and manufactured by Roche NimbleGen. A total of 385 000 unique, overlapping probes 60–90 nucleotides in length were designed including all known exons and untranslated regions (UTRs). In total, 1564 exons from 93 distinct target genes (NCBI build 36.1, hg18) were selected, targeting 845212 bases. The genes had been selected according to our previous gene expression data and their relevance in MDS with ring sideroblasts, and included, for example, *SLC25A37*, *SLC25A38*, *ALAD* or *ABCB6*. 39 of them were related to iron and mitochondrial metabolism. Approximately 5 μg of genomic DNA from 6 MDS patients were fragmented to a size range of 300–500 base pairs (bp) with the use of a GS Nebulizer Kit (Roche Applied Science) to generate blunt-ended fragments. The fragmented DNA was purified (DNA Clean & Concentrator-25, Zymo Research) and analyzed on an Agilent Bioanalyzer 2100 DNA Chip 7500 according to the manufacturer's instructions. The fragmented DNA was then processed according to the recommended NimbleGen protocol (Roche Applied Science, User Guide 3.1; July 2008). In brief, linkers were ligated to the polished fragments in the library to provide a priming site for post-enrichment amplification of the eluted fragment pool. The linker-terminated fragments were then denatured to produce single-stranded products. The resulting library was hybridized to a custom 385K array for 72 h at 42ºC, with the use of the NimbleGen Sequence Capture Hybridization System 4. The hybridized DNA from the target regions was washed and eluted with the use of a NimbleGen Wash and Elution Kit according to the manufacturer's instructions. The eluted sample was amplified by ligation-mediated PCR with the use of primers complementary to the sequence of the adaptors.

**454 Sequencing**

We applied NGS technology using 454 FLX Titanium chemistry according to the manufacturer’s protocols (Roche Applied Science) [1] . Sequencing-compatible linkers were ligated to the eluted samples from the capture microarrays. The libraries were subsequently diluted, amplified on beads using emulsion PCR and sequenced using the 454 FLX sequencing instrument.

**Sequencing data analysis**

Basic raw data analysis was carried out using the GS Run Browser and GS Reference Mapper software version 2.0.01 (Roche Applied Science). Following in silico removal of the linker sequence, each sequence read was compared with the entire appropriate version of the human genome. Captured sequences mapped uniquely back to regions within the target regions were considered sequencing hits. These were then used to calculate the percentage of reads that did hit target regions, and the fold sequencing coverage for the entire target region. All putative variances were first compared with published single nucleotide polymorphism (SNP) data (dbSNP build 130; <http://www.ncbi.nlm.nih.gov/projects/SNP>). We used a custom-made data analysis pipeline to annotate detected variants with various kinds of information, including known single-nucleotide polymorphisms (SNPs), amino acid consequences, genomic location and miRNA binding sites.

**Coverage statistics**

According to NCBI build 36.1, hg18 reference genome, the final target bases covering the target regions were defined to be 845 212 bp; of those 750 594, target bases (99.39%) were covered by capture oligonucleotides as defined by NimbleGens default settings for probe selection. 5 134 bp (0.6%) of the initial target region were omitted due to reasons of specificity and uniqueness. This was sufficient to reach average target coverage of 21.7- fold per individual.

**Reference List**

1. Margulies M, Egholm M, Altman WE, Attiya S, Bader JS, et al. (2005) Genome sequencing in microfabricated high-density picolitre reactors. Nature 437:376-80.
